# Improvement of Kinect_TM_ Sensor Capabilities by Fusion with Laser Sensing Data Using Octree

**DOI:** 10.3390/s120403868

**Published:** 2012-03-26

**Authors:** Alfredo Chávez, Henrik Karstoft

**Affiliations:** Århus School of Engineering, Århus University Finlandsgade 22, 8200 Århus N, Denmark

**Keywords:** sensor fusion, laser, Kinect_TM_, 3D octree map, collaboration

## Abstract

To enhance sensor capabilities, sensor data readings from different modalities must be fused. The main contribution of this paper is to present a sensor data fusion approach that can reduce Kinect_TM_ sensor limitations. This approach involves combining laser with Kinect_TM_ sensors. Sensor data is modelled in a 3D environment based on octrees using a probabilistic occupancy estimation. The Bayesian method, which takes into account the uncertainty inherent in the sensor measurements, is used to fuse the sensor information and update the 3D octree map. The sensor fusion yields a significant increase of the field of view of the Kinect_TM_ sensor that can be used for robot tasks.

## 1. Introduction

Fusion of sensory information is essential in the field of mobile robots. The former is necessary in order to achieve full autonomy and consequently widen the range of its applicability. In this context, it is also necessary to develop more reliable systems which can operate in structured and unstructured environments. The result of the fusing process from the sensory information can be used to reconstruct the environment of the robot, and the robot can plan its own path and avoid obstacles. The robot can also adapt to unexpected environments. In other words, in the process of building the map by fusing sensory information of different sources, a more reliable map is obtained. Therefore, if the mobile robot is suddenly facing unexpected situations in the environment, e.g., people moving around, the robot can update the map taking into account the new entities. Consequently, fusion of different sensor readings must be applied in the hierarchical architecture of the robot.

When dealing with sensor data fusion, one of the requirements to take into account is the choice of the internal representation. This internal representation must be chosen so that it is common to all sensors. This means that sensor readings of different modalities must be converted to the common internal representation in advance before the fusion process is carried out. Occupied as well as empty areas of any arbitrary environments must also be modelled without a prior knowledge of it. It must also represent the estimation and the certainty values of the confidence of the true parameters. The fusion process for different sensors must be feasible under this internal representation. Conversion of sensor data from the physical measurements to the internal representation should be easy to carry out. In this context, the map should be expanded as needed and must have multiple resolution for different mobile robot tasks.

Over the years, several approaches for modelling 3D environments have been proposed. Wurm *et al.* [1] makes a proper review of the previous techniques and also propose a 3D internal representation that fulfils the above requirements. This approach is the OctoMap, which is a library that implements a 3D probabilistic occupancy grid mapping approach.

It is worth mentioning the importance of 3D models for mobile robot tasks. A 3D model has for instance manifold features and can therefore facilitate the disambiguation of different places. Another important fact is that when a mobile robot has to be used in rescue actions and a 3D model of the environment has to be known in advance before any action is taken [2].

The Kinect_TM_ sensor from Microsoft has become quite utilised and has recently become very popular in various mobile robot tasks. However, the narrow field of view and the close range are limitations of the Kinect_TM_. The depth image on the Kinect_TM_ has a field of view of 57.8°. To this end, a good field of view is important in mobile robots, because the wider the field view, the more precise the map, e.g., the robot can catch more features from the environment in a single sensor reading. On the other hand, a mobile robot with poor field view must constantly maneuver to fill up the missing map. One possible solution to this problem is to add one more Kinect_TM_ to increase the field of view. This approach has the disadvantage, however, of dealing with an increase of data and thus becoming a computational burden. Another solution is to rotate the Kinect_TM_ sensor by means of a servo. This again may limit the robot's ability to scan local maps successfully. The minimum range of the Kinect_TM_ is about 0.6 m. This limited range might be a problem when navigating. More precisely, the robot may crash with objects that are situated between the Kinect_TM_ sensor and the minimum range.

The main contribution of this paper is to focus on the problem of fusing range readings from a laser device with a depth Kinect_TM_ image in order to increase the field of view and reduce the minimum range of the Kinect_TM_ sensor. The Hokuyo *U RG —* 04*LX* — *UG*01 laser range finder [3] was selected because of its size and price. It has a sensing range from 0.06 m *→* 4 m. Measurement accuracy is within ±3% tolerance of the current reading for most of the sensors range. The scanning rate is 100 milliseconds across a 240° range. These specifications make the laser ideal for this research in indoor applications.

**Figure 1.**
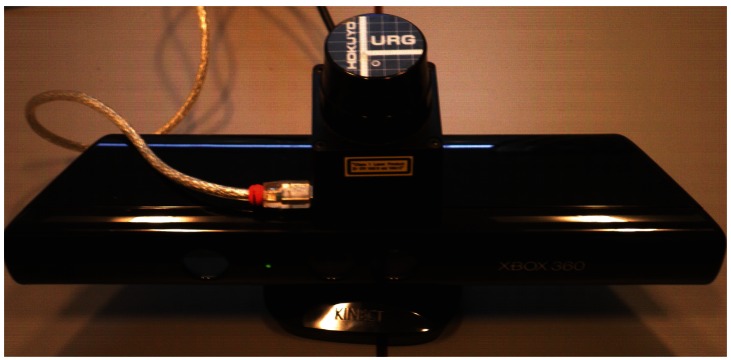
System setup which consists of the Microsoft Kinect_TM_ sensor and the *U RG* – 04*LX* – *U G*01 laser range finder.

The current system setup, as shown in Figure 1 serves as an experimental testbed. It provides data by a Hokuyo laser range finder and a Microsoft Kinect_TM_. Section 2 is concerned with the octree representation. Section 3 describes how the binary Bayes filter can be applied to the octree map in order to fuse and update sensor readings. Section 4 shows the results of the fusion process. Finally, Section 5 gives the conclusion and future research direction.

## 2. 3D Map Making Based on Octree

**Figure 2.**
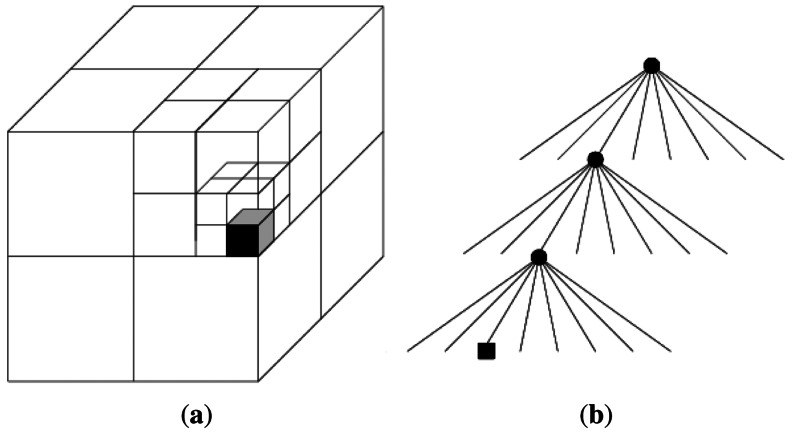
**(a)** The cube has been subdivided into tree depths, where the black cube represents an occupied voxel; **(b)** Octree representation.

Octrees are the three-dimensional generalisation of quadtrees [4]. In other words, an octree is a hierarchical data structure for spatial subdivision in 3D. They have been successfully used to represent 3D maps [1,5–8]. It mainly consists of recursively subdividing the cube into eight octants. Each octant in every division represents a node. The process ends when a minimum voxel size is reached. Figure 2 shows a single occupied voxel and its octree representation.

Sensors suffer from inaccuracies due to noise, hence uncertainties inherited in sensor data readings must be interpreted in a probabilistic fashion. The approach presented in [1] offers a means of combining the compactness of octrees that use discrete labels with the adaptability and flexibility of probabilistic modelling. For this reason, this paper has taken the previous approach.

## 3. Sensor Fusion

Range sensor readings are modelled by probability sensor functions [9] and binary Bayes filter is used to update the occupancy grid [1,7,10,11]. It is mainly used when the state is both static and binary. Equation (1) presents the Odds form of the filter, whereas Equation (2) represents the logOdd (*L*) ratio.

(1) $$\frac{P(n|z_{1:t})}{1-P(n|z_{1:t})}=\frac{P(n|z_{t})}{1-P(n|z_{t})}\frac{P(n|z_{1:t-1})}{1-P(n|z_{1:t-1})}\frac{1-P(n)}{P(n)}$$

(2) $$l_{t}(n)=L(n|z_{1:t})=L(n|z_{t})+L(n|z_{1:t-1})-L_{o}(n)$$

*P*(*n|z_1:t_*) is the probability of a leaf node *n* being occupied given the sensor measurements *z_1_*_:t_*. P*(*n|z_t_*) is the inverse sensor model. The term $L_{o}(n)=\log\left(\frac{P(n)}{1-P(n)}\right)$ is the prior probability of the node and it also defines the initial belief before processing any sensor measurement, e.g., *P(n) =* 0.5. It mainly represents how the distribution of the node is given by an observation. The probabilities *P*(*n|z_1:t_*) can be recovered from the logOdds radio as stated in Equation (3).

(3) $$\begin{array}{ccc} P(n|z_{1:t})=1-\frac{1}{1+\exp\{l_{t}(n)\}} & \text{with}: & l_{t}(n)=\log\left(\frac{P(n|z_{1:t})}{1-P(n|z_{1:t})}\right) \end{array}$$

A new sensor reading introduces additional information about the state of the node *n*. This information is done by the inverse sensor model *P*(*n*|*z_t_*) and it is combined with the most recent probability estimate stored in the node. This combination is done by the binary Bayes filter readings *z_1:t_* = (*z*_t_,…, *z*_1_) to give a new estimate *P*(*n*|*z_t_*). It is worth noting that when initialising the map, an equal probability to each node must be assigned. In other words, the initial node prior probabilities are *P*(*n*) = 0.5.

## 4. Experimental Results

The experiments presented in this work was done using real world data. Moreover, the experiment results verify the problem formulation stated in the introduction, that is, the problem of increasing the field of view and reducing the minimum range of the Kinect_TM_ sensor. In other words, this approach demonstrates that by fusing the Kinect_TM_ with laser sensor data sets, the Kinect_TM_ improves its field of view as well as its minimum close range detection.

**Figure 3.**
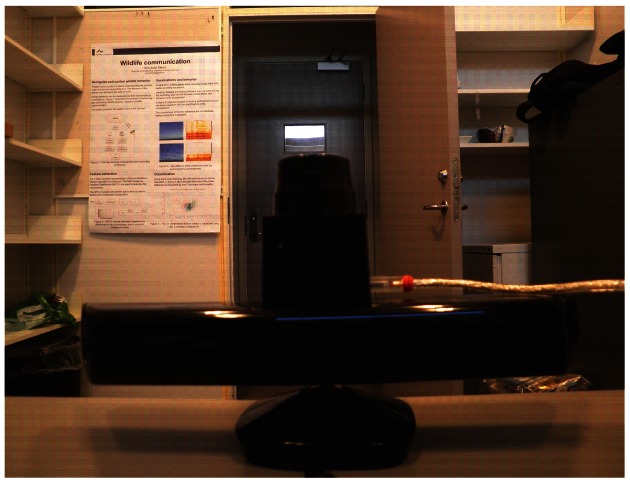
The environment seen by the Kinect_TM_ and the laser range finder, which is placed on the top of the Kinect*_TM_*.

The system setup shown in Figure 1 is used to run the simulation, which results are shown in this section. During the simulation, two indoor data sets from the same environment were recorded using two different sensors. Later on, these two data sets are fused to get a single representation of the 3D scenario. The environment together with the sensor system is shown in Figure 3.

**Figure 4.**
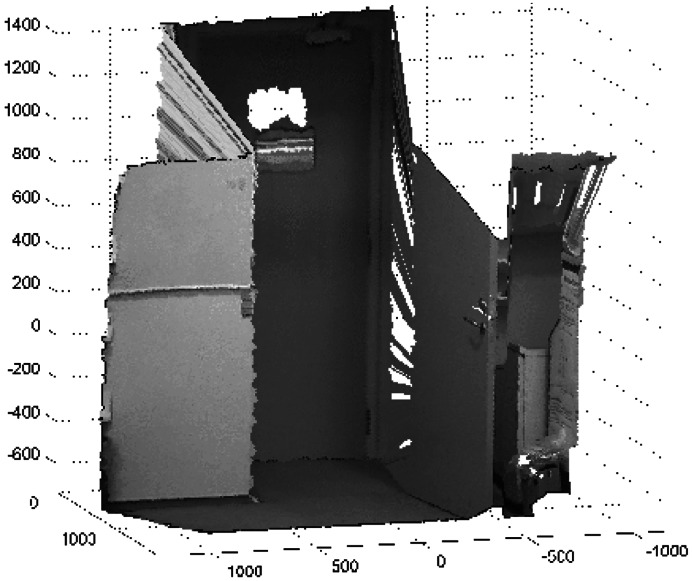
The depth image from the Kinect_TM_ sensor. The units are represented in *mm*.

The first data set was recorded using the Kinect_TM_ sensor. In order to get the Kinect_TM_'s depth image from the sensor, the Openni_TM_ [12] framework libraries were installed in Windows 7. Moreover, the Kinect_TM_ Matlab [13] framework is used to get the 3D (*X, Y, Z*) coordinates from the depth image. Figure 4 visualises the depth image, which resolution is (640 × 480) pixels.

The second data set was recorded using a Hokuyo *URG* – 04*LX* – *UG*01 laser range finder, which is placed on top of the Kinect_TM_ sensor, as seen in Figure 3. By means of the laser driver [14], laser measurements can be obtained. Each single measurement consists of a total of 682 laser scans and are taken over a range of 240°. Each scan represents the Euclidian distance (d) from the center of the laser to the detected object. 2D (*X, Y*) laser coordinates can be obtained using a mapping function *f: d* → (*X,Y*).

Each previous recorded data set is represented probabilistically in a 3D occupancy map by means of the OctoMap library [1]. Moreover, this library is also used to handle the fusion process between these two 3D representations. The library is implemented in C++ and installed on Debian GNU/Linux 6.0.3 (squeeze), released on 8 October 2011.

**Figure 5.**
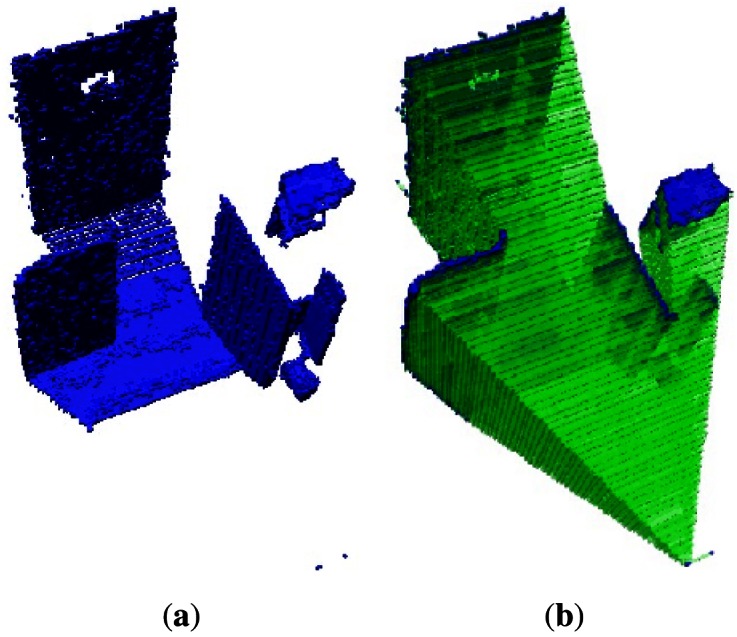
**(a)** First data occupied set volumes of the environment; **(b)** First data empty set volumes of the environment.

A 3D octree map representation of the environment from the first data set that corresponds to the Kinect_TM_ sensor is shown in Figure 5(a). For clarity, only the occupied volumes, which resolutions are 0.2 m, are shown in this Figure. Figure 5(b) shows the empty volumes. The narrow field of view of the Kinect_TM_'s depth image can clearly be seen.

**Figure 6.**
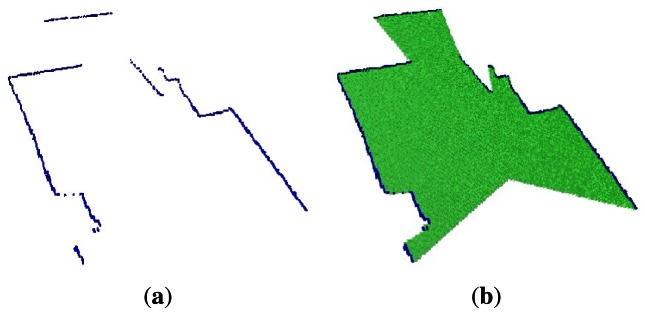
A 2D laser slide of the environment. **(a)** shows the occupied volumes; **(b)** shows the empty volumes.

The second data set represents a 2D slide of the environment, which is represented as an occupied octree maps, shown in Figure 6(a), whereas Figure 6(b) shows the empty and occupied voxels. The main feature of this plot is the well-known wide field of view of the laser.

**Figure 7.**
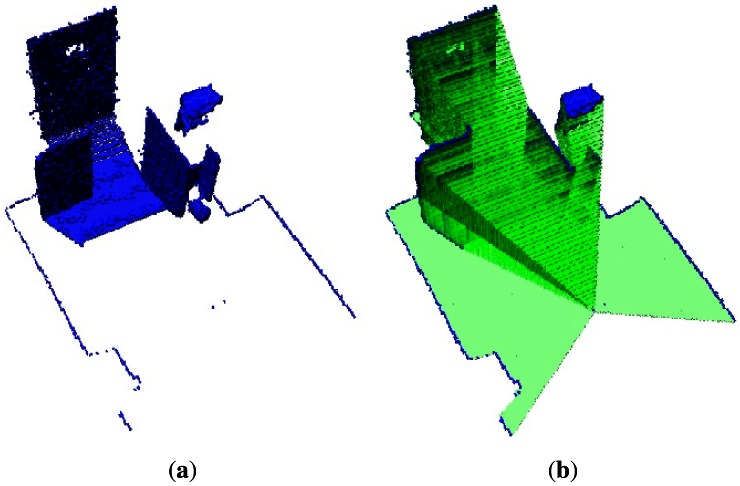
Shows the increased field of view of the Kinect_TM_ sensor. **(a)** Two fused occupied volumes data sets; **(b)** Two fused empty volumes data sets.

The occupied voxels that correspond to the fusion of the two data sets are shown in Figure 7(a). This shows that fusion of sensory information from different sources can increase sensor reliability, in this case by enhancing the field of view of the Kinect_TM_ sensor. The empty volumes are depicted in Figure 7(b). This Figure clearly shows that the robot may have more confidence in its side space. This fact helps the mobile robot to avoid constantly maneuvering to get the missing map, and it can easily react if there is an obstacle in the vicinity of the robot that is not detected by the Kinect_TM_ sensor, but by the laser.

**Figure 8.**
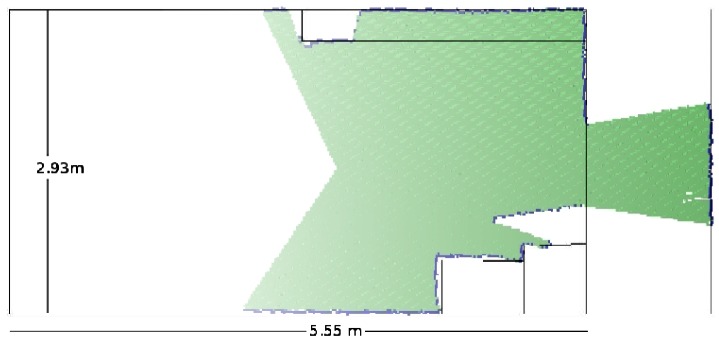
The laser range readings are compared with the true map.

The laser octree data set representation is compared with the true map as shown in Figure 8. The walls, the objects, the corridor and the door are very well detected. This result just confirms the good accuracy tolerance of the current reading for most of the sensor's range.

**Figure 9.**
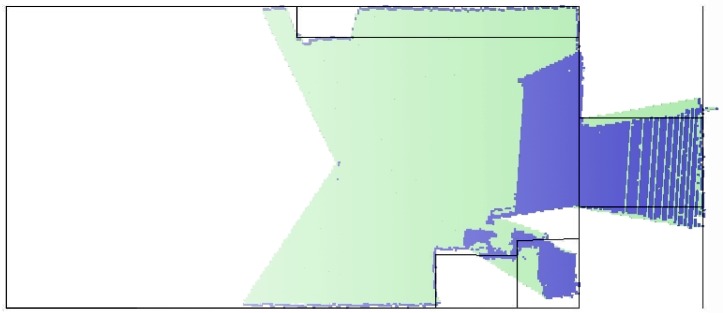
The two fused data sets are compared with the true map.

A 2D slide representation of the two octree fused data sets are also compared with the true map—this can be seen in Figure 9. This result shows the accuracy of the fused maps when compared with the actual environment's map. What is important to notice in this simulation is how the two sensors complement each other. This is achieved as mentioned previously by increasing the poor field of view of the Kinect_TM_ sensor.

**Figure 10.**
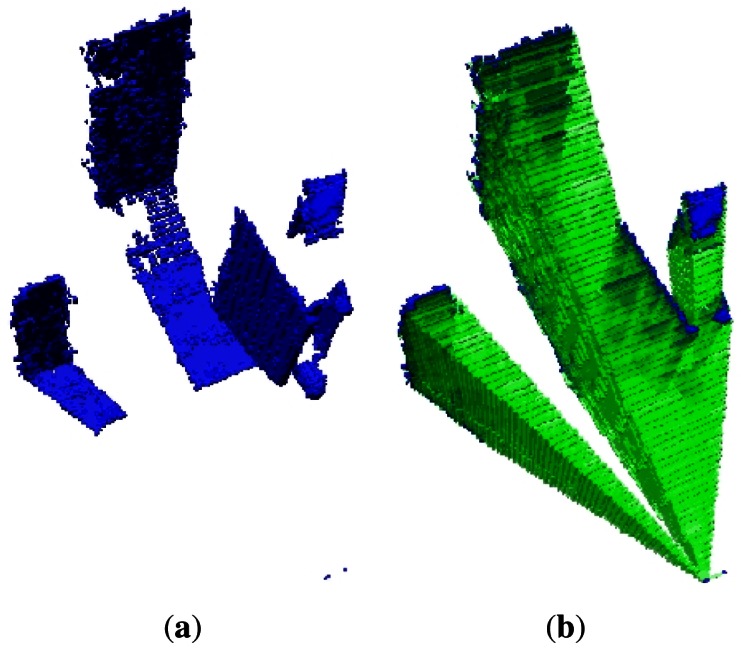
The obstacle is not detected because it has been placed before the minimum range detection of the Kinect_TM_ sensor.

**Figure 11.**
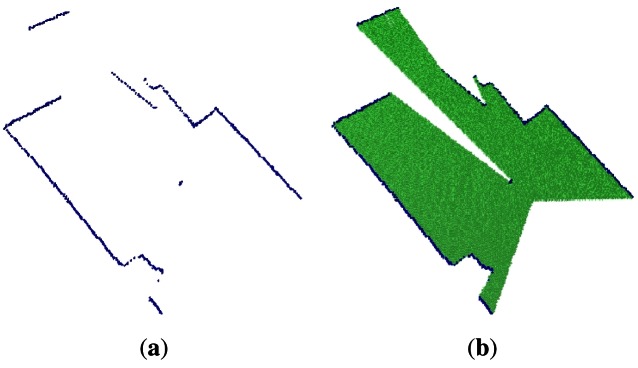
The obstacle is detected because it has been placed after the minimum range detection of the laser sensor.

In order to test the minimum close range, an object has been placed 38*cm* in front of the testbed. This object is placed after the minimum close range detection of the laser, but it is situated before the minimum close range detection of the Kinect_TM_, which means that the object is between the two minimum range detections. The outcome of the Kinect_TM_'s simulation is depicted in Figure 10. It can clearly be seen that the object is not detected due to the mentioned minimum close range limitations of the Kinect_TM_ sensor. However, the laser can detect the object as it was expected, and as shown in Figure 11.

**Figure 12.**
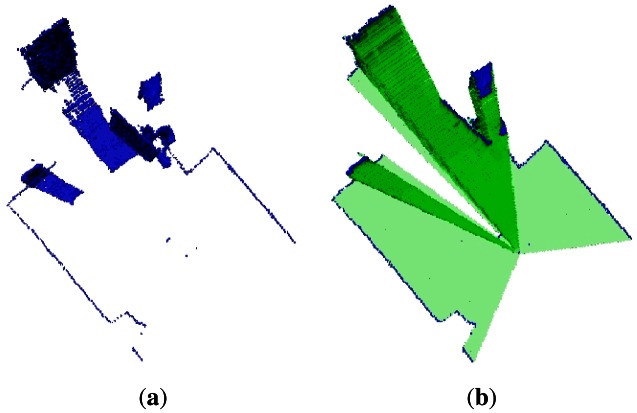
Improvement of the minimum range detection of the Kinect_TM_ sensor.

The fusion of the two previous data set readings is presented in Figure 12. The important fact to be noticed in this simulation result is that the laser really improves the minimum close range detection limitation of the Kinect_TM_ sensor. In doing so, the robot can react and avoid an obstacle that is close and that is not detected by the Kinect_TM_, making the obstacle avoidance and hence the navigation safer and more reliable.

## 5. Conclusions and Future Research

It is very rare that a single sensor can provide sufficient information for the reasoning component. In this sense, the current research in this paper has been focusing on fusing information from two different sources in order to increase the capabilities of a single sensor. To this end, the fusion of a laser readings with features extracted from a depth image using the Kinect_TM_ sensor has come up with good results. It can be observed in Figures 7 and 12 that the two limitations of the Kinect_TM_ sensor, which are (a) the poor field of view and (b) the close range, are overcome by the fusion process. The field of view increments significantly and the close range is reduced; hence objects can be detected closer.

It is believed that the approach of fusing data provided by a laser range and the depth image constitutes an appropriate starting point for a new framework for mobile robots, which tasks of combining the Kinect_TM_ with other sensors are demanding.

A starting point of this framework could be experiments of a dynamic fused 3D map of the environment, where sensor transformation frames are taken into account in order to build the map with respect to a world reference frame. The previous successful results can be used for localization and navigation. It is also the intention of this research to investigate further the applicability of the framework to the combination of different sensors for mobile robot nonlinear control tasks.
